# Composition of the rumen microbiome and its association with methane yield in dairy cattle raised in tropical conditions

**DOI:** 10.1007/s11033-024-09381-0

**Published:** 2024-03-27

**Authors:** Priscila Fregulia, Roberto Júnio Pedroso Dias, Mariana Magalhães Campos, Thierry Ribeiro Tomich, Luiz Gustavo Ribeiro Pereira, André Luis Alves Neves

**Affiliations:** 1 Laboratório de Protozoologia, Instituto de Ciências Biológicas, Universidade Federal de Juiz de Fora, Juiz de Fora, Minas Gerais 36036-900 Brazil; 2 Programa de Pós-graduação em Biodiversidade e Conservação da Natureza, Instituto de Ciências Biológicas, Universidade Federal de Juiz de Fora, Juiz de Fora, Brazil; 3 Brazilian Agricultural Research Corporation, Empresa Brasileira de Pesquisa Agropecuária, EMBRAPA, National Center for Research on Dairy Cattle, Embrapa Gado de Leite, Juiz de Fora, Minas Gerais 36038-330 Brazil; 4 Department of Veterinary and Animal Sciences, Faculty of Health and Medical Sciences, University of Copenhagen, Grønnegårdsvej 3, Frederiksberg C, DK-1870 Denmark

**Keywords:** Greenhouse gases, Enteric methane, Microbiota

## Abstract

**Background:**

Methane (CH_4_) emissions from rumen fermentation are a significant contributor to global warming. Cattle with high CH_4_ emissions tend to exhibit lower efficiency in milk and meat production, as CH_4_ production represents a loss of the gross energy ingested by the animal. The objective of this study was to investigate the taxonomic and functional composition of the rumen microbiome associated with methane yield phenotype in dairy cattle raised in tropical areas.

**Methods and results:**

Twenty-two Girolando (F1 Holstein x Gyr) heifers were classified based on their methane yield (g CH_4_ / kg dry matter intake (DMI)) as High CH_4_ yield and Low CH_4_ yield. Rumen contents were collected and analyzed using amplicon sequencing targeting the 16 and 18S rRNA genes. The diversity indexes showed no differences for the rumen microbiota associated with the high and low methane yield groups. However, the sparse partial least squares discriminant analysis (sPLS-DA) revealed different taxonomic profiles of prokaryotes related to High and Low CH_4_, but no difference was found for protozoa. The predicted functional profile of both prokaryotes and protozoa differed between High- and Low CH_4_ groups.

**Conclusions:**

Our results suggest differences in rumen microbial composition between CH_4_ yield groups, with specific microorganisms being strongly associated with the Low (e.g. Veillonellaceae_UCG − 001) and High (e.g., *Entodinium*) CH_4_ groups. Additionally, specific microbial functions were found to be differentially more abundant in the Low CH_4_ group, such as K19341, as opposed to the High CH_4_ group, where K05352 was more prevalent. This study reinforces that identifying the key functional niches within the rumen is vital to understanding the ecological interplay that drives methane production.

**Supplementary Information:**

The online version contains supplementary material available at 10.1007/s11033-024-09381-0.

## Introduction

The global population is increasing rapidly, and as a result, there is a need to increase the current production of milk and meat by 73% by the year 2050 to meet the growing demand for food. In addition, humanity faces the critical challenge of mitigating the escalating greenhouse gas emissions (GHGs) to reduce the impact of climate change [1, 2].

The rumen of cattle is inhabited by a complex microbiota that plays a crucial role in facilitating the process of rumen fermentation. This process is vital and involves a diverse array of anaerobic microorganisms working together to break down dietary components that are otherwise indigestible by the host animal [3]. This microbial fermentation of feedstuffs in the rumen is responsible for providing the animal with up to 70% of its energy requirements [4]. However, it is important to note that rumen fermentation also results in the production of methane (CH_4_), a GHG that is 28 times more potent than CO_2_ [5]. The livestock industry has been identified as a significant contributor to CH_4_ emissions, accounting for 11% of the total GHGs produced globally, with ruminant emissions playing a dominant role [6, 7]. Instead of being metabolized by the animal, the CH_4_ generated during the rumen fermentation is excreted into the atmosphere through respiration and eructation, resulting in an energy loss of 2–12% [8]. Therefore, there is an urgent need to understand the mechanisms of CH_4_ production in the rumen with the goal of enhancing cattle feed efficiency and mitigating CH_4_ emissions from livestock.

The studies correlating rumen microbiome and methane production are predominantly developed in cattle from temperate climates [9]. Brazil is one of the biggest milk producers in the world [10], and Holstein x Gyr is one of the most common hybrids of the country, since it combines the high milk production from Holstein, the adaptation of Gyr to tropical conditions and the greater tolerance to ecto and endoparasites. The crossbreds have a higher milk production and are more adapted to tropical climates when compared to the pure breeds [11, 12].

Methanogen archaea are of paramount importance in the production of CH_4_ within the rumen. These microbes reduce CO_2_, methanol, or methylamines to form CH_4_ through three distinct metabolic pathways, namely methylotrophy, hydrogenotrophy and acetoclastic [13–15]. While all methanogens aim to generate energy via CH_4_ synthesis, the various rumen methanogenic lineages differ in their metabolic and physiological capabilities. This diversity in physiology and metabolic capabilities can impact the rate of CH_4_ formation in the rumen, with certain lineages potentially contributing to greater CH_4_ emissions than others [15]. The contribution of other rumen microbes to methanogenesis is also significant, such as the symbiotic interplay between ciliated protozoa and methanogens. The protozoan-methanogen interaction is of significant interest because rumen protozoans produce hydrogen, which is used by archaea as a substrate for methanogenesis [14, 16, 17]. This symbiotic interplay within the rumen is of utmost importance and needs to be carefully studied to develop effective strategies for reducing GHG emissions from ruminants. We hypothesized that animals that display varying methane yield values also harbor distinct rumen microbial communities, which can be attributed to specific interactions among different microbial groups that facilitate methane production. The present study aimed to examine the relationship between different rumen microbial groups and their functions in animals classified as high- and low-methane yield in crossbred cattle raised in tropical conditions.

## Materials and methods

The animal procedures used in this study were approved by the Ethics Committee of Embrapa Dairy Cattle (number: 05/2015). The experiment was conducted at the Embrapa Dairy Cattle Experimental Farm, Multiuser Laboratory for Livestock Bioefficiency and Sustainability, located in Coronel Pacheco, Minas Gerais, Brazil.

The present work is a constituent part of an extensive study that aims to gain a comprehensive understanding of the biological parameters associated with feed efficiency and methane production in Girolando cattle – F1 Holstein x Gyr [18–23]. Ornelas et al. (2019) provides a detailed description of the calculation and ranking of methane production, yield and intensity for the animals used in this study.

Briefly, thirty-three F1 Holstein x Gyr heifers were used, with an average body weight (BW) of 293 ± 21.5 kg and an average age of 258 ± 20 d (mean ± SD) at the beginning of the metabolism study. Heifers were housed in individual tie stalls measuring 2.5 × 1.2 m with rubber mats (WingFlex, Kraiburg TPE GmbH & Co., Waldkraiburg, Germany). The diets were composed of 437 g/kg dry matter (DM) and 178 g/kg crude protein (CP) and were comprised of 75% corn silage and 25% concentrate (96% soybean meal and 4% mineral premix, DM basis). Rumen liquids were collected using a stomach tube equipped with a rumen vacuum sampler, immediately snap-frozen using liquid nitrogen, and stored under − 80 °C until further analysis.

The animals were subjected to gas exchange chambers to enable the computation of methane yield. Individual animal gas exchanges were monitored using open-circuit respiratory chambers (No Pollution Industrial Systems Ltd., Edinburgh, UK) equipped with a data acquisition system (Sable Systems International, Las Vegas, USA). Methane measurements were made as described by [24]. The animals were divided into three groups, with eleven animals in each group, based on the CH_4_ data recorded in the respiration chambers (CH_4_ yield (g/kg DMI)). These groups were named high CH_4_ yield (High_CH_4_), medium CH_4_ yield (Medium_CH_4_), and low CH_4_ yield (Low_CH_4_), as described by Ornelas et al. (2019). Since our study aimed to analyze the difference between High and Low CH_4_ yield, only these two extreme groups were used in our analysis (High CH_4_ yield (24.5 ± 0.9 g/kg DMI) (average ± SD) and Low CH_4_ yield (17.7 ± 1.2 g/kg DMI).

In order to explore the rumen microbiome, total DNA was extracted from 2 mL of rumen fluid sample using bead-beating and phenol-chloroform extraction methods (adapted [25]). Briefly, 2 mL of rumen fluid were transferred to a new tube and washed with 1 mL of lysis buffer (500 mM NaCl; 50 mM Tris-HCl, pH 8.0, 50 mM EDTA, 4% SDS). Two µl RNase were added, and the tubes were incubated at 37° C for 15 min. Then, 20 µl of proteinase K were added to the tubes and the cells were lysed by physical disruption using bead beating with a BioSpec Mini Bead-Beater (BioSpec, Bartlesville, OK, USA) at 4,800 rpm for 4 min. The supernatant was transferred to a new tube for phenol-chloroform-isoamyl alcohol extraction. The DNA was precipitated with ammonium acetate 10 M and cold 100% isopropanol. The tubes remained for 30 min in the refrigerator at 4° C and were centrifuged at 16,000 x *g* for 10 min. The supernatant was removed and cold 70% ethanol was added. The tubes were centrifuged at 16,000 x *g* for 2 min. The supernatant was removed and the content was resuspended in 200 µl of TE buffer. The NanoDrop spectrophotometer (NanoDrop Technologies, Inc., Wilmington, DE) and Qubit Quantification Platform (Invitrogen Ltd., Paisley, UK) were used to assess the DNA quantity and quality of the DNA extracted.

Amplicon library preparation (*n* = 22) was implemented by PCR amplification of the V4 region of the 16S rRNA gene of bacteria and archaea, using the primers 515 F (5′-Adaptor/ GTGCCAGCMGCCGCGGTAA) and 806R (5′-Adaptor/GGACTACHVGGGTWTCTAAT) [26]; and by the amplification of the V3-V4 region of the 18S rRNA gene of protozoa, using the primers 316 F (5′-Adaptor/GCTTTCGWTGGTAGTGTATT) and 539R (5′-Adaptor/CTTGCCCTCYAATCGTWCT) [27]. DNA-sequencing library preparation was done using Illumina TruSeq kit following manufacturer’s instructions, then libraries were sequenced on the IlluminaHiSeq2500 sequencing platform (Illumina, Inc., San Diego, CA, USA).

For the bioinformatic analysis, the Quantitative Insights Into Microbial Ecology 2 (QIIME 2) v. 2020.8 [28] was used to analyze sequencing data. The data were demultiplexed, and the sequence reads were quality-filtered, denoised, and merged following the default parameters. The Divisive Amplicon Denoising Algorithm (DADA2) plugin in QIIME2 was used to remove chimeric sequences, and the sequences were truncated at 180 bases to remove low-quality regions. Then, the amplicon sequencing variants (ASVs) table was generated [29]. Representative sequences were aligned to the SILVA 132 Small Subunit rRNA Database for bacteria [30, 31]. The classifier was pretrained on the Silva 18S rRNA database (release 132) for protozoa using the *fit-classifier-Naive–Bayes* method from the *q2-feature classifier* plugin, using the default parameters from QIIME2 *version* 2020.8. The package Phylogenetic Investigation of Communities by Reconstruction of Unobserved States 2 (PICRUSt2) in QIIME2 [32] was used to predict metabolic pathways from ASVs. Fungi taxa were excluded from the analysis since the 18S rRNA molecular marker is unsuitable for their classification.

For the statistical analysis, the sequence count of all samples was standardized by rarefying them to the same number of sequences (the smallest sampling size – 15,234 for 16S rRNA and 166,255 for 18S rRNA). Despite its limitations, rarefaction consistently increases the statistical power to detect differences in alpha and beta diversity metrics [33]. To investigate the Alpha-diversity metrics, we used the default parameters on QIIME2 *version* 2020 to calculate Faith’s Phylogenetic Diversity (PD), Evenness and Shannon’s diversity. Beta-diversity metrics were determined by the weighted UniFrac distance, Jaccard index and Bray–Curtis dissimilarity index. To test the dissimilarity of the samples, we used unweighted UniFrac distance matrices and performed Permutational Multivariate Analysis of Variance (PERMANOVA) with 999 permutations.

The multivariate method implemented in the mixMC (mixOmics microbial community) R package [34] was used to identify specific associations, or signatures, between microbial profiles and functions, and the methane yield phenotype using sparse partial least square discriminant analysis (sPLS-DA). In this analysis, we used the ASV table and the taxonomic table generated from QIIME2, and only microbial taxa and microbial functions with a relative abundance greater than 0.01% and prevalent in at least 50% of the samples (11 out of 22) were considered. The optimal number of components on the sPLS-DA analysis was based on the overall error rate per component. Then, sPLS-DA selected the most discriminative ASVs to identify the microbial signatures associated with the methane yield phenotype, and a supervised analysis was used for selection of discriminative ASVs with sPLS-DA. For the pathway mapping into categories, we used KEGG: Kyoto Encyclopedia of Genes and Genomes (https://www.genome.jp/keg/) (Table 1). The KEGGs were selected according to the highest classification on the Brite Hierarchies.

**Table 1:** KEGG Brite classification. The predicted KEGGs for 16S rRNA and 18S rRNA mapped into the Kyoto Encyclopedia of Genes and Genomes

KEGG	Category
16S rRNA	
K06608	Genetic information processing
K06610	Signaling and cellular processes
K02103	Genetic information processing
K10125	Environmental Information Processing
K18197	Metabolism
K19242	Genetic information processing
K00533	Metabolism
K09948	Not Included in Pathway or Brite
K19341	Environmental Information Processing
K19342	Signaling and cellular processes
18S rRNA	
K01727	Metabolism
K12292	Environmental Information Processing
K10677	Metabolism
K05352	Metabolism
K07488	Genetic information processing
K04127	Metabolism
K02597	Metabolism
K16915	Environmental Information Processing
K12293	Environmental Information Processing

## Results

**Fig. 1:**
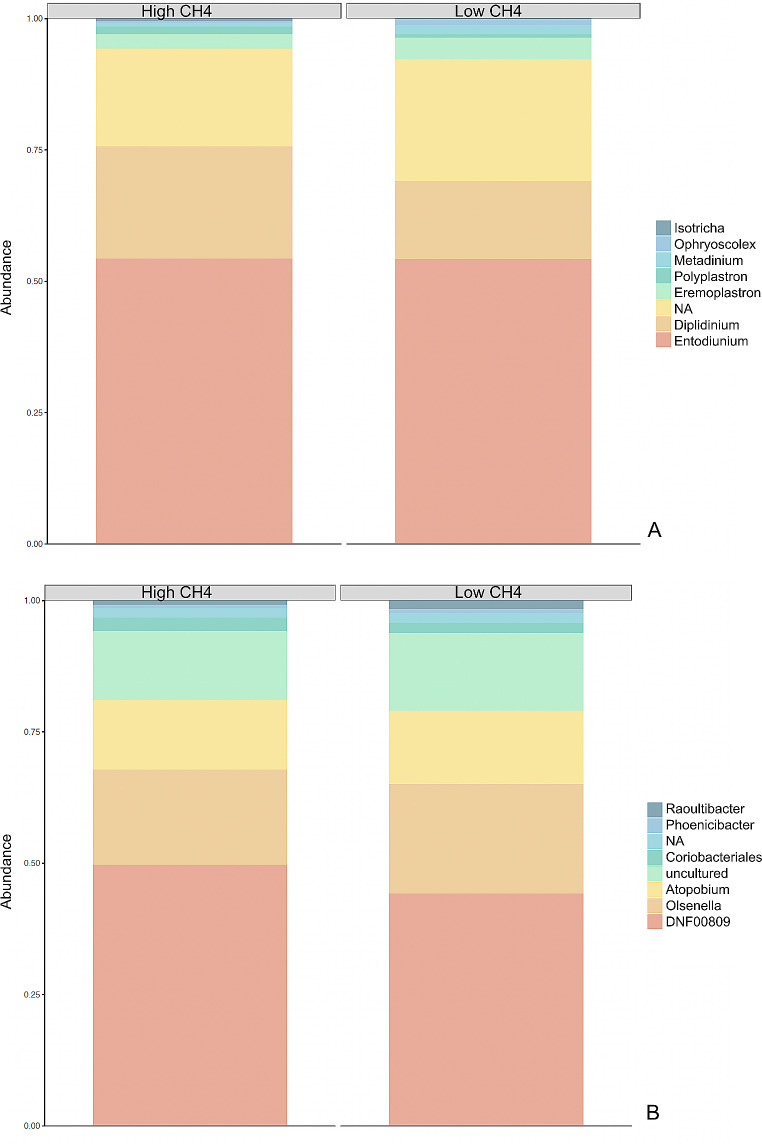
Taxa summary barplot between the methane yield groups (High and Low CH_4_). **(A)** Protozoa. **(B)** Prokaryotes

In total, twenty-two dairy heifers were used to obtain rumen samples, from which 7,019,894 sequences of 16S rRNA reads and 7,216,804 sequences of 18S rRNA reads were generated. The Good’s coverages for both 16 and 18S rRNA were found to be higher than 96% (Table 2). The microbial taxonomic composition associated with each methane yield group is shown in the Fig. 1, Supplementary Table S1.

**Table 2:** Alpha-diversity and Beta-diversity statistics of the rumen microbiota in High and Low CH_4_ in dairy cattle. Significance determined at *p* ≤ 0.05

Diversity metric	Prokaryotes	Protozoa
Faith’s Phylogenetic Diversity	0.76	0.41
Simpson’s Evenness	0.41	0.86
Good’s coverage	96%	99%
Unweighted UniFrac	0.87	0.99

The present study used alpha- and beta-diversity indexes to investigate the relationship between the rumen microbial community and the methane yield phenotype. Alpha-diversity analysis involved calculating Faith’s Phylogenetic Diversity (PD), Evenness, and Shannon’s diversity. For beta-diversity, unweighted UniFrac was used. None of the diversity metrics showed a significant difference between the methane yield groups. Additionally, neither weighted UniFrac, Jaccard index, nor Bray–Curtis dissimilarity matrix showed clustering between the divergent methane yield groups (Table 2). The package PICRUSt2 in QIIME2 was used in order to predict the most abundant microbial functions on the rumen of animals divergent for methane yield. From the 16S rRNA reads and 18S rRNA reads, 7,699 and 7,620 MetaCyc pathways, respectively, were identified (Supplementary table S2). When a microbial taxon is labeled as NA, it means that the sequence was not classified at that particular level.

**Fig. 2:**
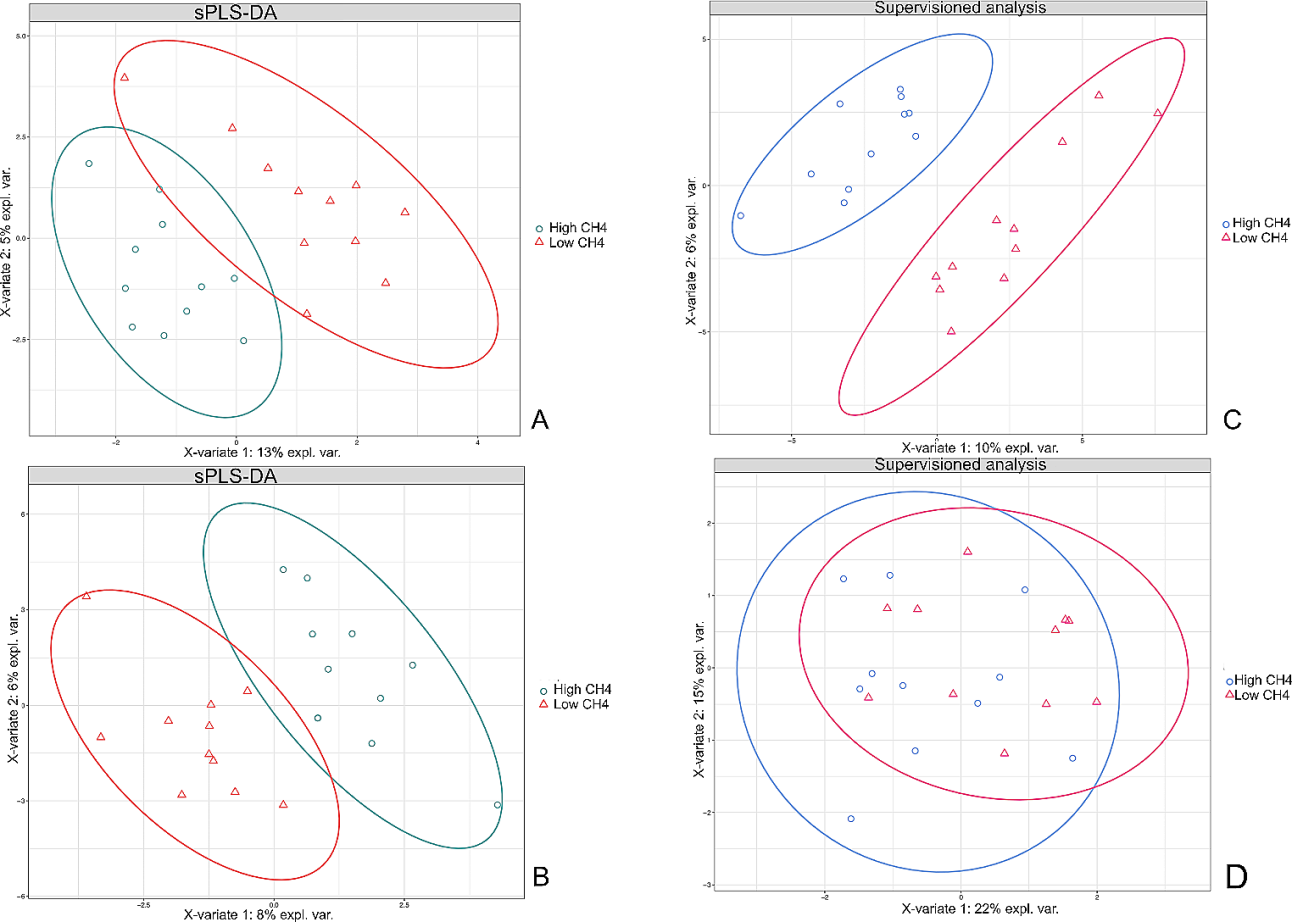
Sparse partial least square discriminant analysis results on rumen microbiome in two FE groups of dairy cattle. Sample plot on the two first sPLS-DA components with 95% confidence level ellipse plots. (**A**) sPLS-DA of prokaryotes; (**B**) sPLS-DA of Protozoa; (**C**) supervised analysis of prokaryotes; (**D**) supervised analysis of protozoa

The sPLS-DA analysis used the ASV table and the taxonomic table from QIIME2 analysis and focused exclusively on microbial taxa and functions that exhibited a relative abundance greater than 0.01%, and were prevalent in at least 50% of the samples (11 out of 22). Following the filtering process and alignment, the ASV were classified into 19 phyla and 149 genera of prokaryotes, and five phyla and ten genera of protozoa (Supplementary table S1). High CH_4_ and Low CH_4_ groups presented different microbial compositions for both 16 and 18S rRNA datasets (Fig. 1). The predicted functional profile from PICRUSt2 resulted in 255 metabolic pathways predicted for 16S rRNA and 255 metabolic pathways predicted for 18S rRNA. The sPLS-DA multivariate analysis revealed that the taxonomic and functional profile for both prokaryotes and protozoa differed between the High- and Low-CH_4_ groups. While there was some overlap between the two groups, most of the microbial functions were exclusive to each methane yield group, as shown in Fig. 2, A-B. In the sPLS-DA supervised analysis of the first two components, there was a clear separation for the taxonomic profile of prokaryotes between the two methane yield groups. However, for the protozoal taxonomic profile, the cluster overlapped, indicating a high degree of similarity in the protozoal community found in animals with high and low methane yield (Fig. 2, C-D).

**Fig. 3:**
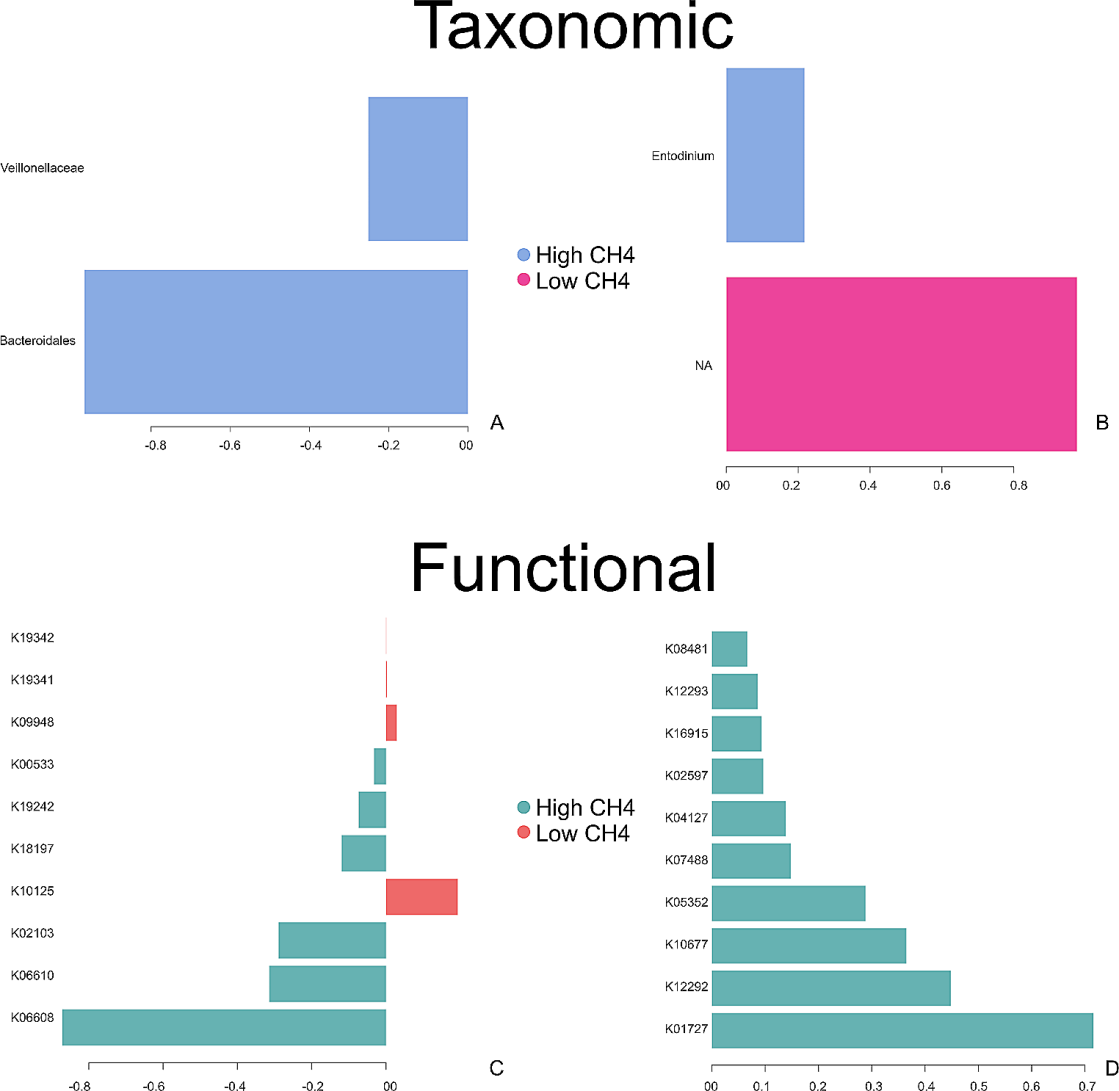
Loading plot of each feature selected on the first component for microbial taxa (**A-B**) and MetaCyc pathways (**C-D**) that best characterize each methane yield group, with color indicating the class with a maximal mean expression value for each microbial taxa or function. X-axis shows the coefficients of selected features and outcomes. (**A**) Prokaryotes, (**B**) Protozoa, (**C**) Prokaryotes, (**D**) Protozoa

The sPLS-DA can select the most discriminative ASVs that best characterize each phenotype group. The ASVs selected on the first component are mostly highly abundant in each group, based on the mean of each ASV per group. For prokaryotes, 100% of the ASVs selected in component 1 characterized the rumen microbiome of the High CH_4_ group, including members classified into the taxa Veillonellaceae_UCG − 001 and Bacteroidales_UCG − 001. For protozoa, 50% of the ASVs selected in component 1 characterized the High CH_4_ group, and it was composed of the taxon *Entodinium*. On the other hand, 50% of the signature characterizing the Low CH_4_ group could not be classified at genus level (Fig. 3, A-B). At the functional level, 60% of the signature selected in component 1 for prokaryotes characterized the rumen functions of the High CH_4_ group, with MetaCyc pathways related to genetic information processing (30%), metabolism (20%), and signaling and cellular processes (10%). In contrast, the functional signature of the Low CH_4_ group comprised environmental information processing (20%), signaling and cellular processes (10%), and one nonclassified pathway (10%). For protozoa, 100% of the functional signature selected in component 1 of the sPLS-DA characterized the rumen functions of the High CH_4_ group, and the functions were related to metabolism (50%), environmental information processing (30%), and genetic information processing (10%) (Fig. 3, C-D).

## Discussion

Cattle exhibit genetic and physical differences in methane production, with a heritability that ranges from low to moderate (h2 = 0.13 to 0.38). This presents an opportunity for the breeding of cattle with lower methane emissions, as research studies have shown [35–37]. Furthermore, it has been observed that animals with a higher feed efficiency are likely to produce less methane [38], but the correlation between feed efficiency and methane production is still unclear [39]. Most breeding programs concentrate on improving feed efficiency alone, and currently, there is no selection for cattle with low methane emissions [40].

The rumen is widely regarded as one of the most diverse ecosystems on the planet, both in in terms of species diversity and functional richness [41]. In previous studies [42], it was found that the methanogenic communities in efficient animals exhibited greater diversity as compared to their inefficient counterparts. Our study showed significant differences in the prokaryotic community between the High- and Low CH_4_ groups (Fig. 2). However, we found no significant difference in the diversity indeces of the rumen microbiota associated with the high and low methane yield groups. This outcome could be attributed to the absence of differences between the groups, or it may be explained by the limitations in the taxonomic classification resolution derived from the amplicon sequencing data used in this study. Moreover, we also observed differences in the metabolic pathways associated with animals that produce high and low levels of methane, for both prokaryotes and eukaryotes. These results are in agreement with the findings of other studies, which suggest that the functional profile of the rumen microbiome is more important to understanding enteric methane production and feed efficiency than the taxonomic profile [39]. This is because microbes from different taxa can utilize similar substrates to produce similar end products [43].

Methane production in the rumen is closely associated with microbial hydrogen production that occurs via the fermentation processes [16]. Recent studies have suggested that microbial substrates, such as hydrogen, play a pivotal role in driving methane production in the rumen [9]. Methanogens in the rumen receive substrates for methanogenesis through bacterial fermentation, which includes hydrogen, carbon dioxide (CO_2_), acetate and methyl compounds. Hydrogen and CO_2_ are the primary substrates for methanogenesis in the rumen [16, 44, 45]. Ciliated protozoa, which are prominent H_2_ producers, maintain a physical association with methanogenic archaea, enabling hydrogen transfer between the two microbes [46]. Thus, a strong correlation exists between bacterial and protozoa hydrogen production and methane formation in the rumen. In this way, the activities of bacterial and protozoal communities contribute significantly to methane yield [16]. While it was previously believed that the abundance of archaea was proportional to CH_4_ production [47], recent studies have shown that the diversity of methanogenic archaea and their interactions with other rumen microorganisms are also essential determinants of the amount of CH_4_ produced [48]. Considering the microbes with the most differential abundance between the groups, we found no clear correlation between any specific microbial taxa or function and the methane yield. Our study further suggests that variations in methane yield are mainly explained by the inter-domain interactions of the rumen microorganisms and their functions, rather than being solely driven by methanogens.

Effective identification of potential mechanisms that influence CH_4_ emissions in livestock production requires a detailed characterization of the functional niches of different microbial groups present in the rumen. Our study demonstrated that the metabolic pathways of prokaryotes and protozoa differ significantly in animals classified as High- and Low CH_4_, and specific microbial taxa are more closely related to each group of methane yield (Fig. 3). Previous research [49] found *Veillonellaceae*, a microbial group that constitutes the core rumen microbiome, to be abundant in low methane-emitting cattle, which differs from our study. Also was found *Veillonellaceae* microbes abundant in low methane-emitting cattle [50]. In contrast, our study discovered a strong association between the Bacteroidales and the High-CH_4_ group. This microbial group is known to contribute to methane production along with archaea [51].

The study suggests that inter-domain associations hold the key to elucidating the mechanisms underlying enteric methane production, rather than relying solely on the examination of archaea composition. We found that specific microbial taxa are differentially related to the methane yield groups (e.g. *Veillonellaceae* and *Entodinium*, respectivelly). The microbial functions also differ on these groups, with a high abundance of metabolic pathways related to metabolism of protozoans in High CH_4_ animals (e.g. K04127, K02597), and more diverse metabolic pathways on the rumen of Low CH_4_ animals, suggesting the relevance of protozoans on the rumen methane production. To gain a comprehensive understanding of the rumen microbiome, it is recommended that meta-omics approaches, such as metagenomic, meta-transcriptomic, and metabolomic analyses, be employed instead of amplicon sequencing. Additionally, the use of single-cell techniques may provide deeper insights into the rumen microbes and their respective roles in rumen fermentation. Despite the limitations of the amplicon sequencing used in this study, specific microbial signatures associated with methane yield phenotype could be defined, which may lead to the development of novel strategies aimed at improving host phenotype for increased sustainability and high productivity.

## Electronic supplementary material

Below is the link to the electronic supplementary material.

Supplementary Material 1

Supplementary Material 2

## Data Availability

The sequence data (16 and 18S) used in the study were deposited on the NCBI website under the BioProject accession number PRJNA1044090. Additionally, all other relevant data pertaining to the study can be found in the supplementary material.
